# Prevalence, awareness, and control of hypertension among Bangladeshi adults: an analysis of demographic and health survey 2017–18

**DOI:** 10.1186/s40885-021-00174-2

**Published:** 2021-09-01

**Authors:** Gulam Muhammed Al Kibria, Rajat Das Gupta, Jannatun Nayeem

**Affiliations:** 1 Department of Epidemiology and Public Health, University of Maryland School of Medicine, Baltimore, MD 21201 USA; 2 Department of Epidemiology and Biostatistics, Arnold School of Public Health, University of South Carolina, Columbia, SC 29208 USA; 3 Chattagram International Dental College and Hospital, Chittagong, Bangladesh

**Keywords:** Hypertension, Awareness, Prevalence, Control, Determinants, Risk factors

## Abstract

**Background:**

The prevalence of hypertension is increasing in Bangladesh, however, few recent studies investigated the proportion of people and factors associated with prevalence, awareness, and control of this condition in this country. This study investigated these among Bangladeshi adults.

**Methods:**

Using Bangladesh Demographic and Health Survey 2017–18 data, a cross-sectional study was conducted. Multilevel logistic regression analysis was employed after descriptive analysis and prevalence estimation.

**Results:**

Among 12,926 persons (mean age: 40 years, 57% women), the prevalence of hypertension was 27.4% (*n* = 3551), it was 28.4 and 26.2% among females and males, respectively. Among hypertensive people, about 42.4% (*n* = 1508) people were aware of having it, 48.7% among females and 33.5% among males. Of the 1313 people who were taking antihypertensive medication, only 33.8% (*n* = 443) had controlled hypertension, 34.7 and 31.7% among females and males, respectively.

Among the studied factors associated with hypertension, people with older age, female gender, overweight/obesity, diabetes, richer wealth quintiles, and residence in some administrative divisions had higher odds of hypertension (*p* < 0.05). However, the odds of awareness was lower among younger people, males, and people without overweight/obesity, diabetes, or richer wealth quintiles. Odds of controlled hypertension was also lower among people with older age and higher among college-educated people.

**Conclusion:**

This study identified several important factors associated with prevalence, awareness, and control of hypertension. It is important to address these factors with nationwide prevention and control programs.

**Supplementary Information:**

The online version contains supplementary material available at 10.1186/s40885-021-00174-2.

## Background

Hypertension is a leading risk factor for cardiovascular disease and a major cause of deaths and disabilities among adults worldwide [1, 2]. Recent trends suggest that the prevalence of hypertension is increasing at an alarming rate among people in low- and middle-income countries (LMICs) [2]. Bangladesh, an LMIC in South Asia, is of no exception from this rising trend [3, 4]. According to the latest Bangladesh Demographic and Health Survey (BDHS) 2017–18, a nationally representative survey from Bangladesh, about 45% of the women and 34% of the men aged 35 years and older are hypertensive in this country [4]. This is a significant rise from the previous estimates of 32 and 20% among women and men of same age group in 2011, respectively [4, 5]. The ongoing demographic transition with an increasing proportion of aging population along with changes in socioeconomic status, lifestyles, and dietary habits of Bangladeshi people could contribute to this higher prevalence [2, 4–6]. This report also shows that more than half of the hypertensive people are not aware of their hypertensive condition [4].

It is important to halt this continues rise in prevalence of hypertension in this country. In addition, to reduce future burden of complications resulting from hypertension, increasing the awareness of hypertension among those who are hypertensive and increasing the control of blood pressure (BP) among those who take any antihypertensive drugs are important [2, 7]. Therefore, identifying the determinants or associated factors of prevalence, awareness, and control of hypertension and addressing them with prevention programs are critical. Using BDHS 2011 data, multiple previous studies reported that people with older age, female gender, urban residence, and higher education level or wealth status had higher prevalence of hypertension [3, 6, 8–10]. These factors may also contribute to awareness and control [7, 11]. However, the proportion of people with prevalence, awareness, or control of hypertension according to background characteristics and factors associated with these three outcomes in recent years are not known. In this study, using a nationally representative sample of Bangladeshi adults, we attempted to estimate prevalence, awareness, and control of hypertension along with the factors that influence these three outcomes.

## Methods

### Study design and setting

We conducted a cross-sectional study by performing secondary analysis on BDHS 2017–18 data. This is the eighth nationwide survey to report major demographic and health indicators on the country. Data collection took place from October 2017 to March 2018. Mitra and Associates, a private research firm in Bangladesh, implemented it [4]. We analyzed the data in January 2021.

A two-stage stratified sample of households was selected. First, for the sampling frame, a list of enumeration areas (EAs) was prepared from the 2011 Population and Housing Census of the People’s Republic of Bangladesh, 250 and 425 EAs from urban and rural areas, respectively. EA was the primary sampling unit and was selected with probability proportional to size. In the EA, a complete list of all the households were done. Then, in the second stage, an average of 30 households were selected from each EA. A total of 20,250 households were selected in this way. From these households, one-fourth of the households were randomly selected for BP and fasting plasma glucose measurements. All adults (i.e., 18 years of age or older) living in these households were invited to participate for measuring BP and fasting plasma glucose. Among 14,704 eligible women and men, about 90% participated. The quality of data collection was monitored by 4 quality control teams from the data collection organization. The questionnaires were translated and validated in the context of Bangladesh. Details of BDHS 2017–18, including survey design, methodologies, sample size calculation, questionnaires, and other statistics are available elsewhere [4].

### Outcome variables

The outcome measures of this study were ‘prevalence (i.e., presence of hypertension) of hypertension’, ‘awareness of having hypertension’ and ‘controlled hypertension among those who take antihypertensive drug’. Systolic BP (SBP) and diastolic BP (DBP) were measured for three times (in millimeters of mercury [mmHg]) with an interval of five minutes between measurements. BP was recorded by digital oscillometric BP device (i.e., LIFE SOURCE® UA-767 Plus BP monitor). Appropriate cuff sizes were used [4]. The mean of the last two measurements was used to classify a respondent as hypertensive. If the third measurement was not available, then the second measurement was used. In case of missing second and third measurements, the first measurement was used. Similar to BDHS 2017–18, we used World Health Organization - International Society of Hypertension cutoffs to classify a person as hypertensive [4, 12]. Therefore, a person with an SBP/DBP of 140/90 mmHg or more was categorized as hypertensive. In addition, participants who reported that they were taking any BP lowering drugs were considered as hypertensive [12].

Among those we found hypertensive, we considered a person as aware if the person knew the diagnosis of hypertension from a doctor or nurse (i.e., “Have you ever been told by a doctor or other health worker that you have high BP or hypertension?”) [4]. Among those who were taking any antihypertensive drugs, we considered ‘controlled hypertension’ if the person had the SBP/DBP below 140/90 mmHg during the measurement of BP.

### Potential determinants/independent variables

Based on published reports and data structure, we investigated age, gender, overweight/obesity, diabetes, education level, wealth quintile, and place and division of residence as potential determinants. Age was categorized into 18–34, 35–44, 45–54, 55–64, and 65 or above years. Overweight/obesity was obtained from body mass index (BMI). BMI was obtained by dividing weight (in Kg) with height (in m^2^). Body weight was measured using a lightweight, electronic SECA 878 scale screen. Height was obtained with a ShorrBoard® measuring board in standing position [4]. Persons with a BMI 23 Kg/m^2^ or more were classified as having overweight/obesity [13, 14]. Fasting plasma glucose was measured using HemoCue 201 RT analyzer [4]. We classified a participant as diabetic if the fasting plasma glucose was 7 mmol/l or more. People who were taking antidiabetic drugs were also considered as diabetic. Participants reported their gender (i.e., male or female) and place (i.e., rural or urban) and division (i.e., Barishal, Chattagram, Dhaka, Khulna, Mymensingh, Rajshahi, Rangpur, or Sylhet) of residence. Education level was categorized into no formal education, primary (i.e., 1–5 school years), secondary (i.e., 6–10 school years), and college or above (i.e., 11 or more school years). Wealth status was obtained from household belongings and basic household construction materials. Similar to previous BDHS, principal component analysis was used to obtain a wealth index score from those components and was then stratified into quintiles: poorest, poorer, middle, richer, and richest [4, 15].

### Statistical analysis

First, we described the study sample with weighted numbers and percentages for categorical variables and means and standard errors (SE) for continuous variables according to the prevalence, awareness, and control status (Tables 1, 2, and 3). To compare continuous variables, we used t-tests and to compare categorical variables, we used chi-square tests. We also reported the prevalence, awareness, and controlled hypertension according to the study variables (Table 4). We have also reported the prevalence among people 30 years of age (Supplemental Table 1). We then obtained the unadjusted odds ratios (UOR) along with 95% confidence intervals (CI) using multilevel logistic regression (Supplemental Table 2). Multilevel regression was done due to the hierarchical nature of the BDHS data (Table 5). Variables with a significance level (i.e., *p*-values) up to 0.2 were investigated into multivariable models to obtain the adjusted OR (AOR) [16]. We obtained variance inflation factors (VIF) with a fake linear regression model to check multicollinearity. We used the provided sample weights to obtain all estimates [4]. Stata 14.0 (College Station, TX, USA) was used to analyze data [17].

**Table 1:** Distribution of the study sample according to presence of hypertension

Variables	Total (*n* = 12,926)	Hypertension		
		Yes (*n* = 3551)	No (*n* = 9375)	*p*-value
Mean systolic BP (SE), mmHg	122.3 (0.25)	145.8 (0.42)	113.5 (0.15)	< 0.001
Mean diastolic BP (SE), mmHg	80.2 (0.14)	91.5 (0.21)	76.0 (0.11)	< 0.001
Age (in years)				
Mean (SE)	39.6 (0.15)	49.0 (0.30)	36.2 (0.17)	< 0.001
18 to 34	5787 (44.8)	738 (20.8)	5049 (53.9)	< 0.001
35 to 44	2585 (20)	735 (20.7)	1850 (19.7)	
45 to 54	1815 (14)	698 (19.7)	1116 (11.9)	
55 to 64	1454 (11.3)	680 (19.1)	775 (8.3)	
65 or more	1285 (9.9)	700 (19.7)	585 (6.2)	
Gender				
Female	7343 (56.8)	2088 (58.8)	5255 (56.1)	0.01
Male	5583 (43.2)	1463 (41.2)	4120 (43.9)	
Diabetes				
Mean fasting plasma glucose (SE), mmol/L	5.7 (0.02)	6.0 (0.05)	5.6 (0.02)	< 0.001
No	10,893 (90.1)	2786 (83.4)	8107 (92.6)	< 0.001
Yes	1199 (9.9)	553 (16.6)	646 (7.4)	
Overweight/Obesity				
Mean body mass index (SE), Kg/m^2^	22.4 (0.06)	23.7 (0.09)	21.9 (0.06)	< 0.001
No	7640 (59.8)	1573 (45.2)	6067 (65.3)	< 0.001
Yes	5129 (40.2)	1904 (54.8)	3225 (34.7)	
Education level				
No formal education	3444 (26.6)	1211 (34.1)	2233 (23.8)	< 0.001
Primary	3839 (29.7)	1047 (29.5)	2792 (29.8)	
Secondary	3790 (29.3)	877 (24.7)	2914 (31.1)	
College or above	1853 (14.3)	416 (11.7)	1437 (15.3)	
Wealth quintile				
Poorest	2459 (19.0)	584 (16.4)	1875 (20)	< 0.001
Poorer	2534 (19.6)	638 (18)	1897 (20.2)	
Middle	2635 (20.4)	705 (19.8)	1930 (20.6)	
Richer	2560 (19.8)	723 (20.4)	1837 (19.6)	
Richest	2738 (21.2)	902 (25.4)	1836 (19.6)	
Place of residence				
Urban	3542 (27.4)	1009 (28.4)	2533 (27)	0.19
Rural	9383 (72.6)	2542 (71.6)	6842 (73)	
Division of residence				
Dhaka	3096 (23.9)	730 (20.5)	2366 (25.2)	< 0.001
Barishal	718 (5.6)	234 (6.6)	484 (5.2)	
Chattagram	2237 (17.3)	666 (18.8)	1571 (16.8)	
Khulna	1586 (12.3)	474 (13.3)	1113 (11.9)	
Mymensingh	1067 (8.3)	252 (7.1)	815 (8.7)	
Rajshahi	1829 (14.2)	503 (14.2)	1326 (14.1)	
Rangpur	1552 (12)	473 (13.3)	1079 (11.5)	
Sylhet	840 (6.5)	220 (6.2)	621 (6.6)	

*SE* Standard error

**Table 2:** Description of study sample according to awareness among those with hypertension

Variables	Total (*n* = 3551)	Awareness		
		Yes (*n* = 1508)	No (*n* = 2043)	*p*-value
Mean Systolic BP (SE), mmHg	145.8 (0.43)	148.0 (0.77)	144.0 (0.42)	< 0.001
Mean diastolic BP (SE), mmHg	91.5 (0.21)	90.8 (0.37)	92.0 (0.21)	0.004
Age (in years)				
Mean (SE)	49.0 (0.30)	52.1 (0.41)	46.8 (0.41)	< 0.001
18 to 34	738 (20.8)	214 (14.2)	524 (25.6)	< 0.001
35 to 44	735 (20.7)	275 (18.3)	460 (22.5)	
45 to 54	698 (19.7)	320 (21.2)	379 (18.5)	
55 to 64	680 (19.1)	361 (23.9)	319 (15.6)	
65 or more	700 (19.7)	338 (22.4)	362 (17.7)	
Gender				
Female	2088 (58.8)	1018 (67.5)	1070 (52.4)	< 0.001
Male	1463 (41.2)	490 (32.5)	973 (47.6)	
Diabetes				
Mean fasting plasma glucose (SE), mmol/L	6.0 (0.05)	6.2 (0.07)	5.9 (0.06)	< 0.001
No	2786 (83.4)	1104 (77.7)	1682 (87.7)	< 0.001
Yes	553 (16.6)	316 (22.3)	237 (12.3)	
Overweight/Obesity				
Mean body mass index (SE), Kg/m^2^	23.7 (0.09)	24.4 (0.13)	23.2 (0.12)	< 0.001
No	1573 (45.2)	572 (38.7)	1001 (50.1)	< 0.001
Yes	1904 (54.8)	905 (61.3)	999 (49.9)	
Education level				
No Formal Education	1211 (34.1)	524 (34.7)	687 (33.6)	0.27
Primary	1047 (29.5)	456 (30.3)	591 (28.9)	
Secondary	877 (24.7)	369 (24.5)	507 (24.8)	
College or above	416 (11.7)	158 (10.5)	258 (12.6)	
Wealth Quintile				
Poorest	584 (16.4)	190 (12.6)	394 (19.3)	< 0.001
Poorer	638 (18.0)	232 (15.4)	406 (19.8)	
Middle	705 (19.8)	295 (19.6)	410 (20.0)	
Richer	723 (20.4)	333 (22.1)	390 (19.1)	
Richest	902 (25.4)	458 (30.4)	444 (21.7)	
Place of residence				
Urban	1009 (28.4)	470 (31.2)	539 (26.4)	0.006
Rural	2542 (71.6)	1037 (68.8)	1505 (73.6)	
Division of Residence				
Dhaka	730 (20.5)	332 (22.0)	398 (19.5)	0.005
Barishal	234 (6.6)	104 (6.9)	130 (6.4)	
Chattagram	666 (18.8)	296 (19.7)	370 (18.1)	
Khulna	474 (13.3)	208 (13.8)	266 (13.0)	
Mymensingh	252 (7.1)	107 (7.1)	145 (7.1)	
Rajshahi	503 (14.2)	196 (13.0)	307 (15.0)	
Rangpur	473 (13.3)	157 (10.4)	316 (15.5)	
Sylhet	220 (6.2)	107 (7.1)	112 (5.5)	

Number are presented along with column percentage unless otherwise specified

**Table 3:** Controlled hypertension status among those taking antihypertensive drugs

Variables	Overall (*n* = 1313)	Controlled Hypertension		
		Yes (*n* = 443)	No (*n* = 870)	*p*-values
Mean systolic BP (SE), mmHg	147.1 (0.82)	121.7 (0.57)	160.0 (0.83)	< 0.001
Mean diastolic BP (SE), mmHg	89.8 (0.39)	79.3 (0.39)	95.2 (0.41)	< 0.001
Age (in years)				
Mean (SE)	52.7 (0.42)	48.7 (0.75)	54.7 (0.51)	< 0.001
18 to 34	163 (12.4)	88 (19.8)	75 (8.6)	< 0.001
35 to 44	236 (17.9)	90 (20.3)	145 (16.7)	
45 to 54	287 (21.9)	91 (20.5)	196 (22.5)	
55 to 64	328 (25.0)	109 (24.6)	219 (25.1)	
65 or more	300 (22.8)	65 (14.7)	235 (27.0)	
Gender				
Female	893 (68.0)	310 (70.0)	583 (67.0)	0.32
Male	420 (32.0)	133 (30.0)	287 (33.0)	
Diabetes				
Mean fasting plasma glucose (SE), mmol/L	6.3 (0.08)	5.9 (0.11)	6.4 (0.10)	0.001
No	948 (76.4)	330 (80.1)	618 (74.6)	0.043
Yes	293 (23.6)	82 (19.9)	211 (25.4)	
Overweight/Obesity				
Mean body mass index (SE), kg/m^2^	24.4 (0.13)	24.5 (0.22)	24.4 (0.16)	0.69
No	486 (37.8)	168 (38.6)	318 (37.3)	0.67
Yes	801 (62.2)	268 (61.4)	533 (62.7)	
Education level				
No formal education	468 (35.6)	129 (29.2)	338 (38.9)	0.007
Primary	395 (30.1)	136 (30.8)	258 (29.7)	
Secondary	320 (24.3)	122 (27.6)	197 (22.7)	
College or above	131 (10.0)	55 (12.5)	76 (8.7)	
Wealth quintile				
Poorest	162 (12.4)	52 (11.7)	110 (12.7)	0.66
Poorer	193 (14.7)	63 (14.3)	129 (14.9)	
Middle	263 (20.0)	84 (18.9)	179 (20.6)	
Richer	289 (22.0)	110 (24.7)	179 (20.6)	
Richest	407 (31.0)	135 (30.4)	272 (31.3)	
Place of residence				
Urban	417 (31.8)	150 (33.9)	267 (30.7)	0.28
Rural	896 (68.2)	293 (66.1)	603 (69.3)	
Division of residence				
Dhaka	297 (22.6)	98 (22.1)	199 (22.9)	< 0.001
Barishal	92 (7.0)	32 (7.2)	60 (6.8)	
Chattagram	273 (20.8)	114 (25.7)	159 (18.3)	
Khulna	173 (13.2)	44 (9.9)	129 (14.8)	
Mymensingh	98 (7.4)	48 (10.8)	50 (5.8)	
Rajshahi	153 (11.7)	34 (7.6)	119 (13.7)	
Rangpur	126 (9.6)	35 (7.9)	91 (10.4)	
Sylhet	101 (7.7)	39 (8.8)	62 (7.2)	

*SE* Standard error

**Table 4:** Prevalence (95% confidence interval) of people with hypertension, awareness, and control

Category	Hypertension	Awareness among hypertensive people	Controlled pressure people taking antihypertensive drugs
Overall	27.4 (26.5, 28.5)	42.4 (40.5, 44.4)	33.8 (30.8, 36.8)
Age (in year)			
18 to 34	12.7 (11.8, 13.8)	29.0 (25.6, 32.7)	54.0 (45.1, 62.7)
35 to 44	28.4 (26.6, 30.3)	37.4 (33.3, 41.7)	38.3 (31.5, 45.6)
45 to 54	38.5 (35.6, 41.4)	45.8 (41.6, 50.0)	31.7 (26.2, 37.8)
55 to 64	46.7 (44.0, 49.5)	53.1 (49.3, 56.8)	33.3 (27.9, 39.2)
65 or more	54.5 (51.5, 57.4)	48.3 (44.4, 52.1)	21.7 (17.5, 26.5)
Gender			
Female	28.4 (27.3, 29.6)	48.7 (46.3, 51.2)	34.7 (31.2, 38.4)
Male	26.2 (24.8, 27.7)	33.5 (30.8, 36.2)	31.7 (27.0, 36.8)
Diabetes			
No	25.6 (24.6, 26.6)	39.6 (37.5, 41.8)	34.8 (31.4, 38.4)
Yes	46.1 (42.7, 49.6)	57.2 (52.4, 61.9)	28.0 (22.7, 34.0)
Overweight/Obese			
No	20.6 (19.5, 21.7)	36.3 (33.8, 39)	34.6 (30.3, 39.2)
Yes	37.1 (35.6, 38.7)	47.5 (45, 50.1)	33.4 (29.7, 37.3)
Education level			
No formal education	35.2 (33.2, 37.1)	43.2 (40.0, 46.6)	27.7 (23.3, 32.5)
Primary	27.3 (25.7, 29.0)	43.6 (40.3, 46.9)	34.5 (29.4, 40.1)
Secondary	23.1 (21.6, 24.7)	42.1 (38.5, 45.8)	38.3 (32.0, 44.9)
College or above	22.5 (20.4, 24.7)	38.0 (33.8, 42.5)	42.1 (33.9, 50.8)
Wealth quintile			
Poorest	23.7 (21.9, 25.7)	32.5 (28.2, 37.1)	32.0 (24.2, 41.0)
Poorer	25.2 (23.1, 27.3)	36.4 (32.3, 40.7)	32.8 (26.2, 40.2)
Middle	26.7 (24.7, 28.8)	41.9 (37.7, 46.1)	31.9 (25.1, 39.5)
Richer	28.2 (26.1, 30.5)	46.0 (42.0, 50.1)	38.0 (31.9, 44.6)
Richest	32.9 (31.0, 34.9)	50.8 (47.0, 54.5)	33.1 (28.5, 38.0)
Place of residence			
Urban	28.5 (26.8, 30.2)	46.6 (43.3, 50.0)	36.0 (31.3, 41.0)
Rural	27.1 (25.9, 28.3)	40.8 (38.4, 43.2)	32.7 (29.1, 36.5)
Division of residence			
Dhaka	23.6 (21.4, 25.9)	45.4 (40.5, 50.5)	32.9 (26.2, 40.5)
Chattagram	29.8 (27.2, 32.4)	44.5 (39.5, 49.6)	41.7 (34.4, 49.4)
Barishal	32.6 (29.5, 35.9)	44.4 (39.0, 50.0)	35.0 (26.5, 44.6)
Khulna	29.9 (27.2, 32.7)	43.9 (38.7, 49.3)	25.4 (19.7, 32.0)
Mymensingh	23.6 (21.2, 26.2)	42.3 (36.0, 48.8)	48.7 (39.7, 57.9)
Rajshahi	27.5 (24.8, 30.4)	39.1 (34.0, 44.3)	22.1 (15.8, 29.9)
Rangpur	30.5 (28.3, 32.7)	33.2 (28.6, 38.2)	27.7 (20.5, 36.3)
Sylhet	26.1 (23.5, 29.0)	48.9 (42.4, 55.4)	38.5 (30.2, 47.5)

**Table 5:** Results of logistic regression analysis to investigate adjusted odds ratio (with 95% confidence interval) for the factors associated with prevalence of hypertension, awareness among hypertensive people, and controlled hypertension among people taking antihypertensive drugs

Variable	Prevalence		Awareness		Controlled hypertension	
	AOR	*p*-value	AOR	*p*-value	AOR	*p*-value
Age (in year)						
18 to 34	Ref. (1.0)		Ref. (1.0)		Ref. (1.0)	
35 to 44	2.7 (2.4,3.1)	< 0.001	1.8 (1.4,2.3)	< 0.001	0.5 (0.3,0.8)	0.003
45 to 54	4.5 (3.9,5.2)	< 0.001	2.8 (2.1,3.6)	< 0.001	0.4 (0.3,0.6)	< 0.001
55 to 64	7.3 (6.2,8.5)	< 0.001	4.2 (3.2,5.6)	< 0.001	0.4 (0.3,0.7)	< 0.001
65 or more	11.9 (10.1,14.1)	< 0.001	4.2 (3.2,5.6)	< 0.001	0.3 (0.2,0.5)	< 0.001
Gender						
Female	Ref. (1.0)		Ref. (1.0)		ND	
Male	0.8 (0.7,0.8)	< 0.001	0.4 (0.3,0.5)	< 0.001	–	
Diabetes						
No	Ref. (1.0)		Ref. (1.0)		Ref. (1.0)	
Yes	1.7 (1.5,1.9)	< 0.001	1.6 (1.3,2.0)	< 0.001	0.7 (0.5,1.0)	0.059
Overweight/Obese						
No	Ref. (1.0)		Ref. (1.0)		ND	
Yes	2.5 (2.2,2.7)	< 0.001	1.5 (1.3,1.8)	< 0.001	–	
Education level						
No formal education	Ref. (1.0)		Ref. (1.0)		Ref. (1.0)	
Primary	1.1 (0.9,1.2)	0.38	1.2 (1.0,1.5)	0.033	1.1 (0.8,1.5)	0.74
Secondary	1.0 (0.9,1.2)	0.59	1.3 (1.0,1.7)	0.024	1.2 (0.8,1.7)	0.38
College or above	1.0 (0.9,1.2)	0.44	1.3 (0.9,1.8)	0.076	1.6 (1.1,2.5)	0.027
Wealth quintile						
Poorest	Ref. (1.0)		Ref. (1.0)		ND	
Poorer	1.1 (1.0,1.3)	0.13	1.3 (1.0,1.8)	0.054	–	
Middle	1.2 (1.0,1.4)	< 0.001	1.5 (1.1,1.9)	0.006	–	
Richer	1.4 (1.2,1.6)	< 0.001	1.7 (1.3,2.3)	< 0.001	–	
Richest	1.4 (1.2,1.7)	< 0.001	1.7 (1.3,2.3)	< 0.001	–	
Place of residence						
Urban	1.0 (0.9,1.2)	0.62	1.1 (0.9,1.4)	0.20	ND	
Rural	Ref. (1.0)		Ref. (1.0)		–	
Division of residence						
Dhaka	Ref. (1.0)		Ref. (1.0)		Ref. (1.0)	
Chattagram	1.5 (1.2,1.8)	< 0.001	1.0 (0.7,1.4)	0.85	1.4 (0.9,2.3)	0.17
Barisal	1.6 (1.3,2.0)	< 0.001	1.1 (0.7,1.5)	0.66	0.9 (0.6,1.6)	0.80
Khulna	1.3 (1.1,1.6)	0.003	1.0 (0.7,1.4)	0.85	0.8 (0.5,1.2)	0.27
Mymensingh	1.1 (0.9,1.4)	0.44	1.0 (0.7,1.4)	0.90	1.6 (0.9,2.8)	0.079
Rajshahi	1.4 (1.2,1.7)	0.001	0.9 (0.6,1.2)	0.48	0.6 (0.3,1.0)	0.061
Rangpur	1.8 (1.5,2.2)	< 0.001	0.8 (0.6,1.2)	0.24	0.7 (0.4,1.3)	0.25
Sylhet	1.4 (1.1,1.7)	0.006	1.5 (1.0,2.1)	0.038	1.2 (0.7,2.0)	0.44

*AOR* Adjusted odds ratio, *ND* Not done, were not adjusted as the unadjusted p-values were more than 0.20

### Ethical approval

The Institutional Review Boards (IRBs) of the ICF International, Rockville, Maryland, USA and Bangladesh Medical Research Council, Dhaka, Bangladesh approved the BDHS 2017–18 protocol. The permission for using the data was obtained in December 2020. All participants provided informed consent to participate into the survey.

## Results

A total of 12,926 participants were included in the analysis (Table 1). Mean SBP and DBP were 122.3 mmHg (SE: 0.25) and 80.2 mmHg (SE: 0.14), respectively. The average age of the participants was 39.6 years (SE: 0.15) and about 56.8% (*n* = 7343) of the participants were females; hypertensive people had a higher proportion of older people or females than people without hypertension. About 9.9% (*n* = 1199) and 40.2% (*n* = 5129) of the participants had diabetes and overweight/obesity, respectively. About 26.6% of the participants had no formal education or 27.4% of the participants were living in urban areas. The overall proportion of people with diabetes, overweight/obesity, or urban residence was higher among people with hypertension than those without hypertension. About one-fourth of the participants were from Dhaka division, 23.9% (*n* = 3096).

Among people who were hypertensive (*n* = 3551), the mean SBP and DBP were 145.8 mmHg (SE: 0.43) and 91.5 mmHg (SE: 0.21), respectively (Table 2). The mean SBP, DBP, age, fasting plasma glucose, and BMI of people with awareness was higher than those without awareness. The proportion of male participants was higher among those without awareness compared to those with it, 47.6% (*n* = 973) and 32.5% (*n* = 489), respectively. The awareness level also differs by wealth status and place or division of residence.

Among those who were taking antihypertensive drugs (*n* = 1313), the mean age and fasting plasma glucose was higher among those who had uncontrolled BP (*n* = 870) than those who had controlled BP (*n* = 443) (Table 3). Among all those who were taking antihypertensive, more than two-thirds were females, 68.0% (*n* = 893), it was similar among those with or without controlled hypertension. About one-fourth had diabetes and two-third had overweight/obesity, 23.6% (*n* = 293) and 62.2% (*n* = 801), respectively. Controlled hypertension level did not differ by wealth status or place of residence, however, it differed by education level and division of residence (*p* < 0.05).

Table 4 shows the prevalence, awareness, and control of hypertension according to background characteristics. The overall prevalence (95% CI) of hypertension was 27.4% (26.5 to 28.5%), it increased with age, 12.7% (11.8 to 13.8%), 28.4% (26.6 to 30.3%), 38.5% (35.6 to 41.4%), 46.7% (44.0 to 49.5%) and 54.5% (51.5 to 57.4%) among people with 18–34, 35–44, 45–54, 55–64, and 65 or more years of age, respectively. Females had a higher prevalence (95% CI) than males, 28.4% (27.3 to 29.6%) and 26.2% (24.8 to 27.7%), respectively. The prevalence also differed according to diabetes, overweight/obesity, education level, wealth status, and place and division of residence. Less than half of the people were aware about hypertensive status, 42.4% (95% CI: 40.5 to 44.4%). More females than males were aware of their hypertensive status, 48.7% (95% CI: 46.3 to 51.2%) and 33.5% (95% CI: 30.8 to 36.2%), respectively. It also differed by overweight/obesity, diabetes, and most other characteristics. About one-third of the people who were taking any antihypertensive drugs had controlled BP, 33.8% (95% CI: 30.8 to 36.8%). It decreased with age. Females had a slightly higher prevalence of controlled BP than males, 34.7% (95% CI: 31.2 to 38.4%) and 31.7% (95% CI: 27.0 to 36.8%), respectively. It also differed by education level and people with a relatively higher education level had a higher prevalence of controlled BP than those with a lower education level.

Table 5 reports the results of multilevel logistic regression to report the factors associated with prevalence, awareness, and control of hypertension. The adjusted odds of having hypertension increased with age. The adjusted odds of hypertension among males were 0.8 (95% CI: 0.7 to 0.8) times the odds among females. Similarly, diabetes (AOR: 1.7, 95% CI: 1.5 to 1.9) and overweight/obesity (AOR: 2.5, 95% CI: 2.2 to 2.7) had significant positive association with hypertension. Factors associated with awareness of hypertension were similar as having hypertension. Older people, people with diabetes or overweight/obesity, higher education level, or wealth status had significantly higher odds of awareness about having hypertension. Lastly, older age was associated with lower odds of controlled hypertension and college or above education level was associated with higher odds of controlled hypertension.

## Discussion

In this study, we observed that more than 1 in 4 people in Bangladesh may have hypertension, however, a majority of them were unaware of having it or did not have controlled hypertension despite taking antihypertensive drugs. We also identified several associated factors for these outcomes, including age, gender, having diabetes and overweight/obesity, education, wealth quintiles, and living in several divisions.

The factors that we observed in these study are similar to the factors identified by previous studies that analyzed BDHS 2011 data, however, the age group of BDHS 2017–18 (i.e., 18 years of age or older) is different than BDHS 2011 (i.e., 35 years of age or older) [3, 4, 8, 18]. Among people with 35–44, 45–54, 55–64, and 65 or more years of age, the prevalence of hypertension was 17.1, 25.0, 30.2, and 40.3%, respectively in 2011, but the prevalence was about 1.5 times high within these age groups in our analysis, 28.4, 38.5, 46.7, and 54.5%, respectively [4, 8]. The awareness level and control remained similar. As mentioned, one major finding is that within less than a decade (i.e., from 2011 to 2017–18), among people with 35 years of age or older, the prevalence rose by more than 1.5 times, this signifies having national prevention and control programs [4]. Bangladesh and many other developing countries are currently facing double disease burden with a simultaneous higher burden of infectious and noninfectious diseases [19]. Multiple non-communicable disease programs were implemented in Bangladesh. However, those programs had inadequate planning, implementation, and monitoring to make them successful [20]. Apart from hypertension, the prevalence of many other chronic diseases (e.g., diabetes and overweight/obesity) has also increased [4]. The substantial rise in prevalence within this short period also indicates a little success of those programs. Therefore, we recommend implementing new prevention and control programs by addressing all the studied factors we identified.

We found positive association of age with prevalence and awareness of hypertension, though it was in opposite direction for hypertension control. Age is a known non-modifiable risk factor for hypertension [21]. With increasing age, structural changes take place in blood vessel walls and the risk for hypertension increases due to stiffness, sodium consumption, lack of dietary potassium, and physical inactivity [21, 22]. Older people also have increased risks for many other chronic conditions such as diabetes, overweight/obesity, chronic kidney disease, and other cardiac diseases that may impact the BP control [3, 21, 23, 24]. These diseases influence the outcome of one another, share many common risk factors, and may cause more severe complications [3, 21]. Overall, all age groups had lower awareness and control levels. Although we found a lower prevalence of hypertension among younger (i.e., less than 35-year-old) people, an overwhelming majority of them did not have awareness about it [4]. Having awareness and control from a relatively younger age are more beneficial to reduce future complications of hypertension [7, 21]. Moreover, adopting healthier lifestyle and dietary habits from younger age may not only reduce the future risks of it but also other chronic diseases mentioned above [21].

Gender was not associated with hypertension control, although the prevalence of hypertension was slightly lower among males, they had significantly lower awareness level. Previous studies in Bangladesh had similar findings [3, 8–10]. Though little is known about gender differences in pathophysiology of hypertension in Bangladesh, studies from other countries suggest differences in physiological and behavioral characteristics may contribute to these differences [25–28].

People with overweight/obesity or diabetes had higher odds of hypertension's prevalence and awareness. In the overall sample, about 25 and 10% people had overweight/obesity and diabetes, respectively. The overall prevalence rates of both conditions have increased in Bangladesh compared to 2011 [4, 18]. This rising trend has been observed in many other developing countries [2, 21, 29, 30]. Halting the rising burden of these conditions would be important not only for reducing hypertension burden but also for reducing the complications arising from themselves. Mortality, morbidity, and health care costs resulting from these conditions are also high [31, 32]. Although the higher awareness among people with diabetes or overweight/obesity could appear as a welcome finding, this finding should be interpreted with caution. As people with diabetes or overweight/obesity may need more health care visits compared to people without these, that could cause this higher awareness among them [33].

We also observed differences in prevalence and awareness according to wealth quintile and place of residence. A higher prevalence of hypertension among people with higher wealth status could be due to the fact that people with a relatively higher wealth quintile in Bangladesh follow more sedentary lifestyle that could contribute to the higher prevalence [9]. The urban-rural differences in prevalence of hypertension was substantially higher in 2011 and urban residents had about 1.5 times higher prevalence than rural people [4]. However, it was minimal in 2017–18 [4]. This indicates that the prevalence is rising rapidly in rural areas compared to urban areas. However, people with a lower wealth status and rural residence have relatively lower awareness level, this difference in awareness may result from differences in health care access, availability, and utilization behavior [4]. More than two-thirds of the people in Bangladesh live in rural regions, therefore, increasing awareness in rural regions is crucial [34]. We also observed some differences in prevalence and awareness of hypertension by division of residence, this may also be due to difference in socioeconomic status and health care utilization behavior according to division of residence [3].

This study has several strengths. First, the survey had a large sample that provided us adequate power for analysis. The survey covered rural and urban regions of all administrative divisions that made the survey nationally representative. BDHS 2017–18 had a high response rate and used standard validated methods that also increased the authenticity of our results [3].

However, limitations of this study also warrant discussion. As this was a cross-sectional study, the overall association may not be causal due to uncertainty about temporal association. Although standard guidelines recommend multiple longitudinal measurements in a clinical setting [21, 35], the BP was measured on a single day, this may have caused some non-differential misclassification. We did not adjust for some other known risk factors (e.g., lifestyle and dietary habits) due to limitations of the dataset [4, 21].

## Conclusions

The prevalence of hypertension has increased but awareness and control level remained lower. To reduce the future burden associated with hypertension, it is essential to address the modifiable factors associated with higher prevalence, lower awareness, and poor control of it by effective awareness and control programs. Reducing prevalence and increasing control of hypertension among older people and males are important. Reducing prevalence of overweight/obesity and diabetes may reduce future burden of hypertension, as well as complications arising from it. Increasing awareness among younger people and prioritizing rural regions to raise awareness is essential.

## Supplementary Information

**Additional file 1: Supplemental Table 1.** Prevalence (95% confidence interval) of people with hypertension, awareness, and control among ≥30-year-old people.

**Additional file 2: Supplemental Table 2.** Results of logistic regression analysis to investigate unadjusted odds ratio (with 95% confidence interval) for the factors associated with prevalence of hypertension, awareness among hypertensive people, and controlled hypertension among people taking antihypertensive drugs.

## Data Availability

Data may be made available upon request to the ICF International, Maryland, USA.

## Abbreviations

AOR: Adjusted odds ratio
BP: Blood pressure
BDHS: Bangladesh Demographic and Health Survey
BMI: Body mass index
DBP: Diastolic blood pressure
LMIC: Low- and middle-income countries
SBP: Systolic blood pressure
SE: Standard errors
UOR: Unadjusted odds ratio
