# Supplementary material for: Prevalence, awareness, and control of hypertension among Bangladeshi adults: an analysis of demographic and health survey 2017–18
Source: Clin Hypertens. 2021 Sep 1;27:17. doi: 10.1186/s40885-021-00174-2 (PMC8408995; doi:10.1186/s40885-021-00174-2)
Supplement: Supplementary file 1 — Additional file 1: Supplemental Table 1. Prevalence (95% confidence interval) of people with hypertension, awareness, and control among ≥30-year-old people. [file 40885_2021_174_MOESM1_ESM.docx]

Supplemental Table 1: Prevalence (95% confidence interval) of people with hypertension, awareness, and control among ≥30-year-old people

| Category | Hypertension | Awareness among hypertensive people | Controlled pressure people taking antihypertensive drugs |
| --- | --- | --- | --- |
| Overall | 35.9 (62.8, 65.4) | 55.1 (53.0, 57.1) | 68.2 (65.2, 71.1) |
| Age (in year) |  |  |  |
| 30 to 44 | 25.1(23.6, 26.6) | 63.2(59.8, 66.5) | 60.2(54, 66) |
| 45 to 54 | 38.5(35.6, 41.4) | 54.2(50, 58.4) | 68.3(62.2, 73.8) |
| 55 to 64 | 46.7(44, 49.5) | 46.9(43.2, 50.7) | 66.7(60.8, 72.1) |
| 65 or more | 54.5(51.5, 57.4) | 51.7(47.9, 55.6) | 78.3(73.5, 82.5) |
| Gender |  |  |  |
| Female | 39.7(38.2, 41.3) | 48.9(46.3, 51.4) | 67.3(63.7, 70.7) |
| Male | 31.5(29.7, 33.3) | 63.9(60.8, 66.8) | 70.2(65.1, 74.9) |
| Diabetes |  |  |  |
| No | 33.9(32.5, 35.2) | 58.1(55.8, 60.4) | 67.6(64.1, 70.9) |
| Yes | 51.5(47.8, 55.1) | 41(36.2, 46.1) | 72.9(66.8, 78.2) |
| Overweight/Obese |  |  |  |
| No | 30.4(29, 31.8) | 59.1(56.5, 61.5) | 69.1(65.3, 72.6) |
| Yes | 49.9(47.6, 52.1) | 48.3(45.1, 51.5) | 66.9(62, 71.4) |
| Education level |  |  |  |
| No formal education | 37(35, 39) | 56.4(53, 59.7) | 72.8(67.9, 77.2) |
| Primary | 34.5(32.5, 36.5) | 54.6(51.2, 57.9) | 67.2(61.6, 72.3) |
| Secondary | 35.4(33, 37.9) | 53.5(49.4, 57.6) | 64.6(57.5, 71.2) |
| College or above | 37.3(33.5, 41.2) | 54.9(49.5, 60.2) | 62.2(53.2, 70.4) |
| Wealth quintile |  |  |  |
| Poorest | 30.6(28.2, 33.1) | 65.6(60.7, 70.2) | 69.2(59.9, 77.1) |
| Poorer | 31.6(28.9, 34.4) | 61.9(57.1, 66.5) | 69.8(62.3, 76.4) |
| Middle | 35.2(32.5, 37.9) | 56.3(51.8, 60.7) | 70.7(63.1, 77.3) |
| Richer | 37(34.2, 39.9) | 49.8(45.4, 54.1) | 63.5(56.7, 69.8) |
| Richest | 44.6(42.1, 47.2) | 46.5(42.7, 50.4) | 68.9(63.7, 73.6) |
| Place of residence |  |  |  |
| Urban | 38.6(36.2, 41.1) | 49.9(46.2, 53.5) | 65.4(60.5, 70) |
| Rural | 34.9(33.4, 36.4) | 57.1(54.6, 59.6) | 69.5(65.7, 73.1) |
| Division of residence |  |  |  |
| Dhaka | 32.5(29.5, 35.7) | 52.1(46.5, 57.6) | 68.2(60, 75.5) |
| Chattagram | 39.3(35.6, 43.2) | 53.2(47.9, 58.4) | 62.1(55, 68.7) |
| Barishal | 41.7(37.9, 45.6) | 53.9(48.1, 59.6) | 67.7(57.7, 76.3) |
| Khulna | 37.9(34.6, 41.3) | 54.7(49.4, 59.8) | 76.7(70.2, 82.1) |
| Mymensingh | 30.1(26.8, 33.7) | 54.8(47.7, 61.8) | 51.7(42.5, 60.7) |
| Rajshahi | 34.7(31.4, 38.2) | 56.7(51.2, 62.1) | 79.7(72, 85.7) |
| Rangpur | 38.4(35.7, 41.1) | 64.6(59.6, 69.3) | 72.4(63.6, 79.7) |
| Sylhet | 34.7(30.8, 38.8) | 48.2(41.6, 54.9) | 63.4(54, 72) |
