# Supplementary material for: Prevalence, awareness, and control of hypertension among Bangladeshi adults: an analysis of demographic and health survey 2017–18
Source: Clin Hypertens. 2021 Sep 1;27:17. doi: 10.1186/s40885-021-00174-2 (PMC8408995; doi:10.1186/s40885-021-00174-2)
Supplement: Supplementary file 2 — Additional file 2: Supplemental Table 2. Results of logistic regression analysis to investigate unadjusted odds ratio (with 95% confidence interval) for the factors associated with prevalence of hypertension, awareness among hypertensive people, and controlled hypertension among people taking antihypertensive drugs. [file 40885_2021_174_MOESM2_ESM.docx]

Supplemental Table 2: Results of logistic regression analysis to investigate unadjusted odds ratio (with 95% confidence interval) for the factors associated with prevalence of hypertension, awareness among hypertensive people, and controlled hypertension among people taking antihypertensive drugs

| Variable | Prevalence | | Awareness | | Controlled hypertension | |
| --- | --- | --- | --- | --- | --- | --- |
|  | UOR | p-value | UOR | p-value | UOR | p-value |
| Age (in year) | | | | | | |
| 18 to 34 | Ref. (1.0) | | Ref. (1.0) | | Ref. (1.0) | |
| 35 to 44 | 2.8 (2.5,3.1) | <0.001 | 1.7 (1.4,2.2) | <0.001 | 0.5 (0.3,0.8) | 0.002 |
| 45 to 54 | 4.4 (3.9,5.0) | <0.001 | 2.5 (2.0,3.2) | <0.001 | 0.4 (0.3,0.6) | <0.001 |
| 55 to 64 | 6.4 (5.7,7.3) | <0.001 | 3.2 (2.6,4.1) | <0.001 | 0.4 (0.2,0.6) | <0.001 |
| 65 or more | 9.3 (8.1,10.6) | <0.001 | 2.8 (2.2,3.5) | <0.001 | 0.3 (0.2,0.4) | <0.001 |
| Gender | | | | | | |
| Female | Ref. (1.0) | | Ref. (1.0) | | Ref. (1.0) | |
| Male | 0.9 (0.8,1.0) | 0.004 | 0.5 (0.4,0.6) | <0.001 | 1.0 (0.7,1.2) | 0.75 |
| Diabetes | | | | | | |
| No | Ref. (1.0) | | Ref. (1.0) | | Ref. (1.0) | |
| Yes | 2.7 (2.4,3.1) | <0.001 | 2.2 (1.8,2.6) | <0.001 | 0.7 (0.5,0.9) | 0.018 |
| Overweight/Obese | | | | | | |
| No | Ref. (1.0) | | Ref. (1.0) | | Ref. (1.0) | |
| Yes | 2.3 (2.1,2.5) | <0.001 | 1.6 (1.4,1.8) | <0.001 | 0.9 (0.7,1.2) | 0.95 |
| Education level | | | | | | |
| No formal education | Ref. (1.0) | | Ref. (1.0) | | Ref. (1.0) | |
| Primary | 0.7 (0.6,0.8) | <0.001 | 1.0 (0.8,1.2) | 0.94 | 1.3 (0.9,1.7) | 0.10 |
| Secondary | 0.5 (0.5,0.6) | <0.001 | 0.9 (0.7,1.1) | 0.28 | 1.5 (1.1,2.1) | 0.01 |
| College or above | 0.5 (0.4,0.6) | <0.001 | 0.8 (0.6,1.0) | 0.025 | 2.1 (1.4,3.1) | <0.001 |
| Wealth quintile | | | | | | |
| Poorest | Ref. (1.0) | | Ref. (1.0) | | Ref. (1.0) | |
| Poorer | 1.1 (1.0,1.3) | 0.093 | 1.2 (0.9,1.6) | 0.12 | 1.0 (0.6,1.6) | 0.94 |
| Middle | 1.3 (1.1,1.4) | 0.001 | 1.5 (1.2,1.9) | 0.001 | 0.9 (0.6,1.5) | 0.81 |
| Richer | 1.4 (1.2,1.6) | <0.001 | 1.7 (1.3,2.2) | <0.001 | 1.1 (0.7,1.6) | 0.79 |
| Richest | 1.8 (1.6,2.0) | <0.001 | 2.2 (1.8,2.8) | <0.001 | 1.1 (0.7,1.6) | 0.69 |
| Place of residence | | | | | | |
| Urban | 1.1 (1.0,1.2) | 0.046 | 1.3 (1.1,1.6) | 0.001 | 1.1 (0.8,1.4) | 0.54 |
| Rural | Ref. (1.0) | | Ref. (1.0) | | Ref. (1.0) | |
| Division of residence | | | | | | |
| Dhaka | Ref. (1.0) | | Ref. (1.0) | | Ref. (1.0) | |
| Chattagram | 1.4 (1.2,1.6) | <0.001 | 0.9 (0.7,1.2) | 0.49 | 1.3 (0.8,2.1) | 0.25 |
| Barisal | 1.6 (1.3,1.9) | <0.001 | 1.0 (0.7,1.3) | 0.88 | 0.9 (0.6,1.5) | 0.68 |
| Khulna | 1.4 (1.2,1.7) | <0.001 | 1.0 (0.7,1.3) | 0.80 | 0.7 (0.4,1.1) | 0.15 |
| Mymensingh | 1.0 (0.8,1.2) | 0.80 | 0.9 (0.6,1.2) | 0.38 | 1.6 (0.9,2.6) | 0.079 |
| Rajshahi | 1.2 (1.0,1.5) | 0.019 | 0.7 (0.5,1.0) | 0.049 | 0.5 (0.3,0.9) | 0.019 |
| Rangpur | 1.5 (1.2,1.8) | <0.001 | 0.6 (0.5,0.9) | 0.003 | 0.7 (0.4,1.2) | 0.18 |
| Sylhet | 1.1 (1.0,1.4) | 0.16 | 1.2 (0.8,1.6) | 0.36 | 1.2 (0.8,2.0) | 0.37 |

UOR: Unadjusted odds ratio
